# Bioprospecting Soil Bacteria from Arid Zones to Increase Plant Tolerance to Drought: Growth and Biochemical Status of Maize Inoculated with Plant Growth-Promoting Bacteria Isolated from Sal Island, Cape Verde

**DOI:** 10.3390/plants11212912

**Published:** 2022-10-29

**Authors:** Catarina Cruz, Paulo Cardoso, Jacinta Santos, Diana Matos, Etelvina Figueira

**Affiliations:** 1 Department of Biology, University of Aveiro, 3810-193 Aveiro, Portugal; 2 Centre for Environmental and Marine Studies, Department of Biology & CESAM, University of Aveiro, 3810-193 Aveiro, Portugal

**Keywords:** plant growth-promoting bacteria (PGPB), maize, drought, sustainability, crop production, arid regions

## Abstract

Climate change and anthropogenic activities are responsible for extensive crop yield losses, with negative impact on global agricultural production. The occurrence of extreme weather events such as drought is a big challenge for agriculture, negatively impacting crops. Thus, methodologies reducing crop dependence on water will be a great advantage. Plant roots are colonized by soil bacteria, that can establish beneficial associations with plants, increasing crop productivity and plant tolerance to abiotic stresses. The aim of this study was to promote plant growth and to increase crop tolerance to drought by inoculation with osmotolerant bacterial strains. For that, bacteria were isolated from plants growing in Sal Island (Cape Verde) and identified. The osmotolerance and plant-growth promotion (PGP) abilities of the strains were determined. A maize seed cultivar tolerant to drought was inoculated with the strains evidencing best PGP capacity and osmo-tolerance. Results evidenced the ability of some bacterial strains increasing the development and inducing osmotolerance in plants. These results evidence the potential of osmotolerant bacteria to further increase the level of tolerance of maize varieties tolerant to drought, decreasing the dependence of this crop on irrigation, and open new perspectives to growth maize in drought affected areas and to use water more efficiently.

## 1. Introduction

According to the United Nations [1] by 2050 the world’s population is expected to increase from 7.87 billion (in 2021) to 9.7 billion, about 23% higher than today and 6% higher than the predicted by FAO [2]. Nowadays, the prevalence of malnutrition worldwide is about 11% and expected to be higher in developing regions in the years to come [1]. Therefore, food production must increase by 70% in order to feed the growing human population, and the production of cereals such as maize, wheat and rice will need to rise to about 3 billion tones [2,3,4]. Maize is a crucial crop to ensure global food security, since it is highly used as biofuel, food source and feed crop globally. In fact, maize is the third most cultivated crop worldwide, and contributes about 62% to the total cereal production, thus any factor impacting maize production will have a serious impact on food security [5,6].

Environmental changes negatively impacts global food production. Climate change has caused inter-seasonal yield variability and negative impact on crop growth, development and production. The major challenges to agriculture are related to climate change severity and occurrence of extreme weather events, such as floods, drought and heat haves but also to agricultural management (application of fertilizers, tillage, agrochemicals) [5,7,8].

Drought is one of the major constraining factors of crop productivity, leading to about 34% of agricultural and livestock production loss in less developed and low-income countries, and has affected more people worldwide than any other environmental stress over the past 50 years [9,10]. Globally, it is estimated that approximately 55 million people are directly affected by drought every year, making this environmental stress the most serious hazard to crops and livestock [11,12]. Due to climate change and reduced precipitation it is expected that droughts will become more frequent and intense in some areas [9,13]. Compared to all the sectors, agriculture is the one more affected by drought, for it sustains 82% of all drought impacts [10]. In fact, recent data shows that, in Europe, drought is responsible for about 9% of cereal yield reduction, and its severity on crop production in the last 50 years nearly tripled [14]. Crops are exposed to several environmental stresses, but drought is considered the most damaging, for it reduces substantially growth, rate of cell division and expansion, stem elongation, leaf size, root proliferation, crop productivity and water use efficiency [6,15,16,17,18]. Drought is also considered the main cause for land degradation, desertification and aridity, and its effects are observed at multiple geographical scales [19]. In fact, it is estimated that about 12 million hectares of land are lost each year due to drought and desertification, and that the percentage of our planet affected by drought in the past 40 years has doubled [12,20]. Drought affects nutrient imbalance, carbon dioxide assimilation in plants, negatively impacting a wide range of physiological and biochemical processes, such as photosynthesis, respiration or enzyme activity [6,17,18]. The impacts of drought depend on occurrence and distribution of rainfall, the capacity of soils to retain moisture and evaporative demands [21,22]. Under drought stress, stomata close partially or totally, and photosynthesis is inhibited [6]. Plants respond to drought with a series of protective adaptations, either morphological, molecular, physiological or biochemical. The responses can be at the whole organism or cellular level. In order to maintain cellular functions under osmotic stress, the production of osmolytes, such as proline (among other amino acids), glycine betaine, polyols and organic acids [15] is crucial.

Due to their sessile nature, plants release a wide range of chemical signals to establish interactions with other organisms, such as nitrogen-fixing bacteria, mycorrhiza and plant growth-promoting bacteria (PGPB) [23,24]. These microbial interactions take place in the rhizosphere, the narrow zone of soil mainly influenced by root secretions [25] where the exudates produced by plant roots are used as the main nutrient and energy source for innumerous microorganisms [23,24]. When it comes to abiotic stress, it was already proven that some soil microorganisms, PGPB, are able to successfully mitigate the negative effects caused by drought in plants [26,27]. PGPB can be free-living, form specific endosymbiosis with plants (such as *Rhizobium*), or be endophytic. The plant growth mechanisms used are similar between these bacteria, and can be divided into direct or indirect mechanisms [28]. Some of the direct mechanisms consist in nitrogen fixation, production of phytohormones (such as auxins and gibberelins), siderophore production and phosphorous solubilization [29]. PGPB also have positive effects by directly inducing systemic resistance (ISR), which protects the plant from pathogenic attacks and increase their resistance to environmental stresses like drought and salinity, by producing osmoprotectants [16,23]. Plant growth can be also promoted indirectly by inhibition of pathogens through competition or antibiosis (biocontrol) [28].

It was described in some studies that inoculation with PGPB can ameliorate osmotic stress in plants like rice [30], wheat [31] and maize [32], mainly through higher nutrient availability. It was also proven that osmotolerant bacteria display higher ability to increase plant tolerance to drought [33,34,35]. Overall, these microorganisms can be utilized as biofertilizers, being more ecofriendly than chemical fertilizers, in promoting plant development and growth under suboptimal conditions [16].

The aim of this study was to evaluate the effectivity of bacterial strains from an arid environment on plant tolerance to osmotic stress. For that, bacterial strains were isolated from Sal Island (Cape Verde), the osmotic tolerance and several growth promotion traits of bacterial strains were screened, and the effect of bacterial inoculation on a drought-tolerant maize variety to osmotic stress was assessed, by evaluating germination, growth and the physiological and biochemical status of plants. The comparison between plants exposed to watered and drought conditions allowed to elucidate the bacterial influence on the biochemistry of plants that contributed to stress alleviation.

## 2. Results

### 2.1. Bacterial Plant Growth-Promoting (PGP) Traits

All strains have the ability to produce IAA, siderophores and alginate (Table 1). Three strains are not able to solubilize phosphate, but the others evidence a moderate solubilization ability (1.04−1.97) with strains H, J, O and U having the higher ratios (1.97, 1.88, 1.94 and 1.88, respectively). Strain S stands out by exhibiting the highest production of IAA, siderophores and alginate, although it is not able to solubilize phosphate. On the other hand, strain K is the highest producer of alginate and IAA.

Eight strains were able to promote seedling emergence under control and osmotic stress conditions: A, D, F, G, Q, R, S and T. Strain O slowered seedling emergence compared to control (non-inoculated seeds) and the remaining strains had a marginal effect (Table 1).

### 2.2. Exposure of Bacteria to PEG

#### 2.2.1. Cellular Damage

**Figure 1:**
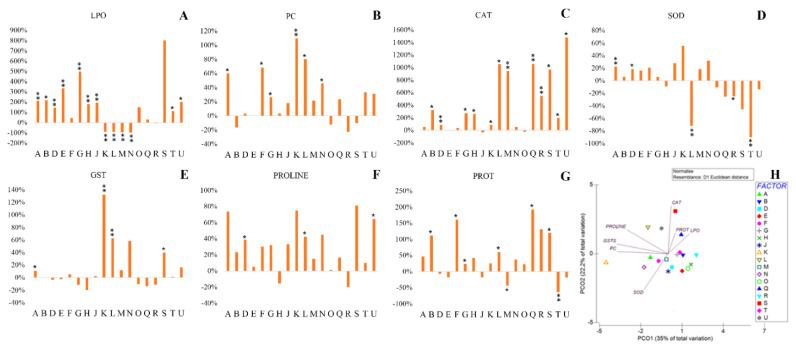
Biochemical parameters evaluated in rhizobacteria isolated from plants grown in Cabo Verde: (**A**) Lipid peroxidation, (**B**) Protein carbonylation, (**C**) Catalase, (**D**) Superoxide Dismutase, (**E**) Glutathione S-Transferase, (**F**) Proline, (**G**) Protein, (**H**) principal coordinates ordination of biochemical parameters under drought stress. Biochemical markers variation of bacterial cells exposed to PEG50 relatively to control (not exposed to PEG). Bacterial strains: *Pantoea* spp. (A); *Klebsiella* spp. (B); *Pseudomonas* spp. (D); *Pseudomonas* spp. (E); *Acinetobacter* spp. (F); *Stenotrophomonas* spp. (G); *Enterobacter* spp. (H); *Enterobacter* spp. (J); *Pantoea* spp. (K); *Pseudomonas* spp. (L); *Rhizobium* spp. (M); *Paenarthrobacter* spp. (N); *Ochrobactrum* spp. (O); *Pseudomonas* spp. (Q); *Rhizobium* spp. (R); *Stenotrophomonas* spp. (S); *Pseudomonas* spp. (T); *Enterobacter* spp. (U). Values are means of three replicates + standard deviation. Significant differences compared to non-inoculated watered plants (control) were marked with single asterisks (*p* < 0.05) or double asterisks (*p* < 0.01). For additional information (means, standard deviation and statistical significance) see supplementary Table S1.

Exposure to osmotic stress significantly increased lipid peroxidation in most bacterial strains (Figure 1A), with G and S showing the highest increases (500% and 800%, respectively). Only 4 strains significantly showed lower LPO levels when exposed to osmotic stress (K, L, M and N).

Osmotic stress also increased the damage in proteins (Figure 1B) in most strains, being the damage significant in 46% of them.

#### 2.2.2. Antioxidant Response

Exposure to PEG increased catalase activity in most strains, significantly in B, D, G, H, K, L, M, Q, R, S, T and U strains (Figure 1C).

Relatively to SOD activity, exposure to osmotic stress only significantly increased SOD in strains A and D and decreased it in L, R and T. In fact, 28% of the bacterial strains had almost no change in SOD activity (Figure 1D).

Glutathione S-transferases activity was poorly influenced by the presence of PEG, with 67% of the strains not evidencing changes in the enzyme activity. K and L significantly increased GST activity (>50%) (Figure 1E).

#### 2.2.3. Osmolyte Production

Out of the 18 strains, 13 were able to increase proline production between 20% and 80% (Figure 1F), significantly in D, L and U.

#### 2.2.4. Protein Content

Half of the bacterial strains were able to increase protein content between 30% and 300% relatively to control (Figure 1G), significantly higher in B, F, G, L, Q and S. On the other hand, M and T showed significant reduction in protein levels, 44% and 63% respectively.

#### 2.2.5. Multivariate Analysis

The PCO was performed with the difference between drought and control, showing the effect of drought in bacteria. In the multivariate analysis (Figure 1H) it is observed that PCO1 explains most of the drought effects on bacterial biochemistry.

Alterations induced by exposure to PEG are highly correlated to the activity of antioxidant enzymes (GSTs and CAT), osmolyte production (proline) and protein carbonylation, with Pearson correlations higher than 0.85. In the negative axes of PCO1 and PCO2 it is possible to observe that strain K is highly influenced by PC, GST and SOD activity. On the other hand, A, F and N evidenced similar responses to drought, with increases of protein carbonylation (protein damage), but were able to trigger the antioxidant response (SOD activity). Being near the origin, M strain displayed low influence of drought on its biochemistry. In the positive axis of PCO2 it is noticeable the high correlation of strains S, L, Q and U to catalase activity (S, Q and U), LPO (S), proline (L and U) and protein content (Q). The positive axis of PCO1 reveals the majority of the strains that increased lipid peroxidation when exposed to drought stress (B, D, E, G, H, J, O and T) and the ones that decreased PC (B and O), GST activity (D, E, G, H, O and R) and proline content (H and R).

### 2.3. Biochemical Response of Plants to Drought

#### 2.3.1. Biometric Parameters

**Figure 2:**
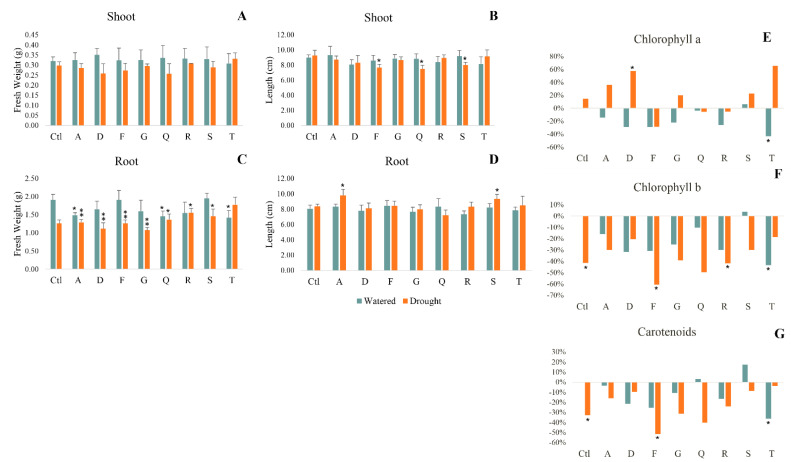
Maize plants grown for 7 days in watered and drought conditions. Morphometric parameters: fresh weight (**A**) and length (**B**) of shoots; fresh weight (**C**) and length (**D**) of roots. Variation of photosynthetic pigments relatively to control (watered and non-inoculated plants): chlorophyll a (**E**), chlorophyll b (**F**) ad carotenoids (**G**). Watered (greenish bars) and drought (orange bars) plants were inoculated with different strains: *Pantoea* spp. (A); *Pseudomonas* spp. (D); *Acinetobacter* spp. (F); *Stenotrophomonas* spp. (G); *Pseudomonas* spp. (Q); *Rhizobium* spp. (R); *Stenotrophomonas* spp. (S); *Pseudomonas* spp. (T). Values are means of three replicates + standard deviation. Significant differences compared to non-inoculated watered plants (control) were marked with single asterisks (*p* < 0.05) or double asterisks (*p* < 0.01). For additional information (means, standard deviation and statistical significance) see supplementary Table S2.

It is observed that the inoculation with bacterial strains did not have a significant effect on shoot growth and biomass both in watered and drought plants (Figure 2A,B).

The influence of bacterial inoculation is more noticeable in plant roots. Under drought, some bacterial strains significantly mitigated the negative impact of drought on root weight (Figure 2C). When inoculated with R and T under stress it is noticed a significant promotion of 24% and 41% (respectively) in comparison with drought control. Root length significantly increased when plants were inoculated with A and S, under drought stress (Figure 2D).

#### 2.3.2. Photosynthetic Pigments

In watered plants, there is a trend to decrease photosynthetic pigments in inoculated plants, significantly reduce when inoculated with strain T (Figure 2E−G). Under drought, inoculation with D significantly promoted chlorophyll a synthesis by 58% (Figure 2E). The effect of drought on chlorophyll b and carotenoids content was mostly negative or had no impact, but inoculation with some strains (A, D, S and T) alleviated the significant negative impact of drought observed in non-inoculated plants (Figure 2F,G).

#### 2.3.3. Cellular Damage

**Figure 3:**
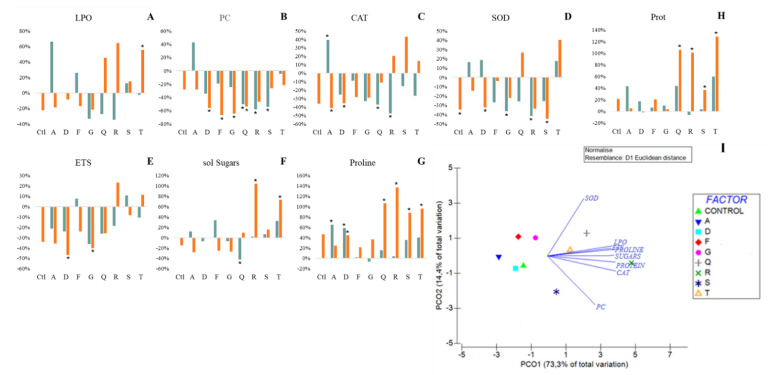
Biochemical parameters evaluated in maize plants grown for 7 days in watered and drought conditions: (**A**) Lipid peroxidation, (**B**) Protein carbonylation, (**C**) Catalase, (**D**) Superoxide Dismutase, (**E**) Electron Transport System, (**F**) Soluble Sugars, (**G**) Proline, (**H**) Protein, (**I**) principal coordinates ordination of biochemical parameters under drought stress. Biochemical markers variation of bacterial cells exposed to PEG50 relatively to control (not exposed to PEG). Watered (greenish bars) and drought (orange bars) plants were inoculated with different strains: *Pantoea* spp. (A); *Pseudomonas* spp. (D); *Acinetobacter* spp. (F); *Stenotrophomonas* spp. (G); *Pseudomonas* spp. (Q); *Rhizobium* spp. (R); *Stenotrophomonas* spp. (S); *Pseudomonas* spp. (T). Values are means of three replicates + standard deviation. Significant differences compared to non-inoculated watered plants (control) were marked with single asterisks (*p* < 0.05) or double asterisks (*p* < 0.01). For additional information (means, standard deviation and statistical significance) see supplementary Table S3.

Most LPO changes due to drought and inoculation were not significant. Inoculation with T increased significantly LPO levels (Figure 3A).

Overall, drought and inoculation decreased PC levels, significantly for Q, R and S in control conditions and for D, F, G and Q under osmotic stress (Figure 3B).

#### 2.3.4. Antioxidant Response

Under control conditions, inoculation led to reduced CAT activity (Figure 3C), significantly for Q and R (by 37% and 47% respectively), exception being made for strain A that significantly increased it. Under drought stress, inoculation with A and D significantly reduced CAT activity.

Under control conditions, inoculation with G and R strains reduced significantly SOD activity (Figure 3D). Exposure to drought decreases significantly SOD activity in non-inoculated plants and plants inoculated with D and S.

#### 2.3.5. Metabolism

Under stress, strains D and G significantly reduced ETS, but overall ETS activity was not significantly affected by drought or inoculation.

Relatively to protein content (Figure 3H), both drought and inoculation led to higher protein, in most plants. Significant increases in protein production are observed in plants inoculated with Q, R, S and T, with increases over 30%.

#### 2.3.6. Osmolyte Production

Overall, the accumulation of soluble sugars (SS) was not significantly affected by drought or inoculation (Figure 3F). Exception being made for Q, R and T that significantly decreased SS content under control conditions (Q) and increased under drought stress (R and T).

On the other hand, drought and bacterial inoculation increased proline content (Figure 3G). Strains A and D significantly increased proline under control conditions (65% and 59% respectively). When exposed to drought, strains D, Q, R, S and T significantly promoted proline production (44%, 107%, 137%, 88% and 96%).

#### 2.3.7. Multivariate Analysis

The PCO shows the effect of bacterial inoculation on the response of plants to drought stress. In the PCO (Figure 3H) it is observed that all parameters have high correlations, ranging from 0.88 to 0.98, with LPO and CAT having the highest values (0.97 and 0.98, respectively). The drought effects on non-inoculated plants (control), and plants inoculated with A and F positioned them on the negative side of PCO1 and PCO2 axes, showing low influence of bacterial inoculation on the biochemical changes plant underwent when exposed to drought. On the positive side of PCO1 and PCO2 axes it is observed the strain that influenced positively most parameters (R). Strains T and Q are close positively influencing mostly SOD and LPO. Strains F and G are positioned in the positive side of PCO2 and on the negative side of PCO1 axes, showing the ability to reduce protein carbonylation and to increase antioxidant response (SOD activity) under stress. S strain is positioned in the positive PCO1 and negative PCO2 axes evidencing the influence of this strain on the increase of PC on drought compared to watered plants. Strain R stands out by being positioned in the most positive value of PCO1 increasing CAT activity, soluble sugars accumulation and protein and proline content in plants as a response to water shortage.

## 3. Discussion

The present study aimed to investigate the effects of osmotic stress on bacteria and maize, the biochemical mechanisms associated, and the contribution of PGPB tolerant to osmotic stress, isolated from an arid environment, the Sal Island in Cape Verde, on plant tolerance to drought. The use of a drought tolerant variety of *Z. mays* allowed to understand the biochemical mechanisms underlying the plant tolerance, and to evaluate if Cape Verde strains can further increase the plant tolerance to drought. Several studies already described the effects of osmotic stress on bacteria [36,37,38], and the contribution of plant growth-promoting bacteria on plant tolerance to drought [39,40,41,42,43,44,45]. Some studies reported the use of drought tolerant bacterial strains to enhance plant growth [33,34,35], showing that osmotolerant strains can be more efficient in drought stress mitigation in inoculated plants.

Following the classification of Sá et al. (2019), six strains were moderately tolerant to osmotic stress (IC_50_ between 7.5% and 10% PEG, that is between −0.13 and −0.19 MPa, according to Guo et al. [46]). The plant growth-promoting traits evaluated showed that all strains are capable of siderophore and IAA production. Siderophores are proven to be one of the most crucial survival strategies for microorganisms, for they form complexes with iron (Fe) and other metal ions, improving their solubility and uptake, which can enhance plant growth and development [33,47,48]. On the other hand, was already reported that higher production of siderophores by bacterial strains is associated with increased drought resistance in inoculated plants [49].

The phytohormone IAA, the most common plant hormone of the auxin class, plays a crucial role in cell division, fruit development and cell elongation [50]. When inoculated with bacteria able to produce IAA, plants benefit from increased root length and density (primary and lateral), having a well-developed root system, imperative for efficient water and nutrient uptake and to anchor plants in the soil [50,51,52]. Besides increasing plant length and biomass, IAA producing bacteria are involved in the induction of tolerance to drought stress, and are reported to boost chlorophyll contents and nutrient uptake [52,53].

Out of the 18 strains, 15 are able to moderately solubilize phosphate. Phosphorus represents a crucial role in vital plant functions, such as growth, sustenance and development. Phosphate solubilization is a plant growth-promoting trait described as the ability to convert unavailable forms of phosphorus into forms easily absorbed by plants, promoting plant growth and yield [54]. Additionally, this PGPB trait was associated with increased drought tolerance in plants [41,55,56,57].

Osmotic stress can cause oxidative damage in cells, resulting from the production of reactive oxygen species (ROS) [58]. ROS can react with metabolic and physiologic important biomolecules causing irreversible damage in lipids, proteins and DNA [59], or even cell necrosis and death [27]. Organisms deal with oxidative stress through different mechanisms that regulate ROS concentrations, for example, by producing ROS scavenging enzymes, such as superoxide dismutase and catalase [27].

When exposed to osmotic stress, a response pattern was observed in the different strains. Overall, osmotic stress led to high protein carbonylation and to high lipid peroxidation, evidencing that both membranes and proteins were negatively affected by osmotic stress, as already reported in other studies (e.g., [36]). As a mechanism to cope with the oxidative stress generated, most strains showed higher SOD and CAT activity. A significant increase in proline content was also observed and helped in the osmoregulation of plant cells and protecting proteins from oxidative damage. Indeed, several studies showed that proline is essential to cellular osmoregulation, and its synthesis under drought conditions is associated with stress tolerance both in bacteria and plants [36,39,59,60,61,62]. The increase in GST activity induced by osmotic stress was also observed in the present study, and evidences that the biotransformation activity was triggered to minimize the toxicity cause by endoxenobiotics produced during stress, further protecting cells from damage [63].

Drought in one of the major causes of agricultural loss. Presently, almost 40% of arable land is in arid and semi-arid regions, being drought a real concern. PGPB are being recognized to play a crucial role in mitigation of drought effects on plants, and the use of bacteria isolated from semi-arid soils is being regard as a promising strategy for increasing plant tolerance to stress, and to reduce the amount of water necessary for irrigation without compromising yields. The results of the present study evidenced that some strains (R and T) induced higher biomass in roots, privileging branching, while others (A and S) induced higher length in drought stressed plants, evidencing the potential increase in water uptake efficiency of these plants, as reported in other studies [64,65]. Given the fact that strains R and T are moderately tolerant to osmotic stress, it corroborates the fact that osmotolerant bacteria are able to increase plant tolerance to drought, by facilitating the absorption of water in conditions of water shortage scarcity, and enabling a significant increasing of plant biomass compared to non-inoculated plants.

As observed in the greenhouse experiment, drought negatively affected the photosynthetic pigments, which may lead to lower CO_2_ fixation [66] and decreased plant growth and productivity. Different studies noticed the positive influence of PGPB on plant photosynthetic pigments content [67,68,69]. In the present study, inoculation with some strains increased the amount of chlorophylls a and b in drought stressed plants, indicating a positive contribution to plant tolerance.

On the biochemical level, inoculation with PGPB induced different responses in maize plants. As mentioned by other studies, protein carbonylation is the most common protein modification caused by reactive oxygen species, being this biochemical parameter used as a marker of oxidative damage in proteins [70,71]. Overall, inoculation reduced protein carbonylation both under drought and control conditions. Half of the strains (A, D, F and G) were able to mitigate drought effects on membranes (LPO decrease). On the contrary Tiepo et al. [72], observed that all the strains used to inoculate neotropical trees (*Cecropia pachystachya* and *Carinianna estrellensis*) increased LPO compared to non-inoculated plants. The decrease in cell damage observed in the present study may be supported by the activity of antioxidant and biotransformation enzymes, which increased the capacity of cells to reduce ROS and to overcame the oxidative stress induced by drought in cells.

Accumulation of osmolytes, as proline and soluble sugars help cells to osmoregulate and reduce the effects of low osmotic potential on cell metabolism, proving to be crucial to both bacteria and plant [58]. In the present study it was observed that inoculation with PGPB markedly increased the proline content in plants, both in drought and control conditions. Similar results with plants exposed to drought stress were observed in other studies, with plant growth-promoting bacteria showing capacity to ameliorate the effects caused by drought, by increasing proline content [41,42,73]. However, in this study the proline content observed in the not-inoculated plants was substantially higher than in those studies, with proline content over 60 mg/g FW in both irrigated and drought conditions. For example, Moreno-Galván et al. [73] recorded proline contents in non-inoculated maize plants around 50 μg/g FW in irrigated plants and approximately 200 μg/g FW in plants exposed to drought. This evidences a constitutive characteristic (production of proline in high amounts) of the maize variety used in our study and that may justify, at least in part, the described drought tolerance.

The amount of soluble sugars on maize plants under drought stress was previously reported, and inoculation with PGPB increased it [39,44,74,75,76,77]. Comparing non-inoculated plants, the maize variety used in this study showed higher ability to accumulate soluble sugars than other maize varieties [39,75], both in watered (around 2 fold) and drought conditions (by around 1.5 fold), evidencing a constitutive ability to accumulate osmolytes. The present study also shows different influences of strains on inoculated plants both in control and drought conditions. While two strains (R and T) were able to significantly increase the accumulation of soluble sugars under drought, three reduced it (A, F and G). Despite the observed reduction in soluble sugars in plants inoculated with strains A, F and G soluble sugars contents were still higher than in maize plants from other studies [39,44,75,76]. Evidencing once more that the ability of plants to accumulate osmolytes, in this case soluble sugars, is important for the tolerance of maize plants to drought.

The increase of protein content in plants under drought stress was already described [39,75,78,79]. It was reported that soluble protein prevents cellular components and molecules decomposition and denaturation, especially under stress [75]. In fact, some studies reported that when plants are inoculated, the content in protein is higher than in non-inoculated plants [39,75,78], corroborating the present results, where 50% of the strains significantly increased protein content. Most of the soluble protein in a cell are enzymes [80]. Thus, an increase in soluble protein means that the metabolism is increased, by induction of metabolic pathways involved in stress coping [81].

Plant mitochondria transform energy into forms, such as the phosphate nucleotide ATP, easily used by cells in metabolic processes crucial for cell adaptation to abiotic stresses. Under drought stress, mitochondria display the ability to defend themselves from excess of reactive oxygen species, through ROS detoxification, repairing ROS mediated damage and by keeping the electron transport chain oxidized [82]. ETS activity in mitochondria measurement provides information about respiratory potential and allows the estimation of plants general metabolic activity [83]. In the present study ETS activity was reduced under water stress by most strains, showing the influence of bacterial inoculation in plant cells energy use. This effect was also observed in other plant species, such as basil [84], Masson’s pine [85] and potato [83].

## 4. Materials and Methods

### 4.1. Bacterial Strains

The bacterial strains used in this study were isolated from two plant species (*Acacia albida* and *Amaranthus viridis*) sampled from Sal Island, Cape Verde (16°35′33″ N–22°55′29″ W). After identification, 18 strains were obtained. The accession number associated to each strain is included in Table 1.

### 4.2. Tolerance to Polyethylene Glycol (PEG)

PEG 6000 was purchased from Tokyo Chemical Industry (TCI) and used as received. According to the manufacturer, this polymer has an average molecular weight around 6000.

The tolerance to drought was determined following the method described by Sá et al. (2019). The strains were inoculated in Yeast mannitol broth (YMB) medium supplemented with peptone and different concentrations of PEG (0, 7.5, 10, 12.5, 15, 17.5, 20 and 25%). The tubes were incubated in an orbital shaker (150 rpm) at 26 °C for 15 h, and the optical density was measured at 620 nm. After determining the PEG concentration that inhibits growth by 50% (IC_50_), each bacterial strain was grown at two osmotic conditions (0% PEG-control and % PEG inhibiting each strain 50%) for 15 h at 26 °C. Optical density was measured, cells were pelleted by centrifugation at 10,000 rpm for 10 min at 4 °C, and stored at −20 °C for further biochemical analysis.

### 4.3. Bacterial Ability to Promote Plant Growth

#### 4.3.1. Siderophore Production

For siderophore production, the methodology described by Arora and Verma [86] was followed with some modifications. The strains were inoculated (100 μL) in YMB medium supplemented with 1 g/L of peptone (control) and incubated for 15 h at 150 rpm in an orbital shaker. After growth, the optical density was measured, and 1.5 mL of growth medium was collected to a microtube. The microtubes were centrifuged for 5 min (12,000 rpm) at 4 °C and the supernatant used for siderophore quantification. Into a 96-well microplate, 130 μL of sample (supernatant), and 20 μL of chrome azurol S reagent (CAS) were pipetted. A blank was prepared with 130 μL of non-inoculated medium and 20 μL of CAS. The optical density was measured at 630 and 750 nm after 20 min incubation. The siderophore production (percent siderophore unit−PSU) was quantified by the formula described by Arora and Verma [86]:$\;\frac{\left(\mathrm{Ar}\;-\mathrm{As}\right)\;\times \;100}{\mathrm{Ar}}$, where Ar stands for the absorbance of the blank and As represents the absorbance of the samples. The results were expressed in PSU/Optical density.

#### 4.3.2. Alginate Production

Alginate produced by bacteria was determined following the method described by Johnson et al. [87], with some modification [36]. Bacteria were inoculated in modified YMB medium supplemented with 1 g/L of peptone for 15 h at 150 rpm in an orbital shaker. After the optical density was measured, 2 mL of each tube was collected to a microtube, centrifuged for 10 min (at 12,000 rpm) at 4 °C, and both pellet and supernatant collected in different microtubes for determination of alginate in two fractions: present in the medium and attached to the cell wall.

In order to determine the alginate present in the medium, after centrifugation 165 μL of the supernatant was transferred to a different microtube, to which was added 35 μL of 517 mM ethylenediamine tetraacetic acid (EDTA). The sample was incubated at 37 °C for 1 h.

The pellet was resuspended in 1 mL of dH_2_O and posteriorly centrifuged, and the supernatant discarded. To the pellet 200 μL of 100 mM EDTA were added and the microtubes were incubated at 37 °C for 1 h in an ultrasonic bath. After incubation of both samples, the microtubes were centrifuged and 100 μL of the supernatant were pipetted to the 96-well plate, followed by 100 μL of 120 μM dimethylmethylene blue (DMMB).

A standard curve was performed in a 96-well microplate to determine the amount of alginate in the samples, alginate diluted in distilled water in concentrations ranging from 0 to 500 μg/mL was used as standard. The absorbance was measured at 525 and 595 nm. The results were expressed in μg/mL/OD.

#### 4.3.3. Indole Acetic acid Production

The methodology to evaluate the indole acetil acid (IAA) production was based on the protocol of Gordon and Weber [88] with some modifications [89]. Initially, 100 μL of inoculum was added to tubes with YMB medium supplemented with 100 μL of tryptophan (5000 μg/mL). The tubes were incubated for 15 h at 150 rpm in an orbital shaker. After growth, the optical density was measured, and 2 mL was collected to a microtube and centrifuged at 10,000 g for 10 min at 4 °C. In a 96-well microplate 100 μL of the supernatant and 200 μL of Salkowsky reagent were added, and after 25 min incubation at room temperature, the absorbance was measured at 530 nm. Two blank wells were performed, one with 100 μL of YMB medium with tryptophane and 200 μL of Salkowsky reagent and the other one with 100 μL of YMB medium without tryptophane and 200 μL of Salkowsky reagent.

A standard curve was performed (0 to 40 μg IAA/mL) and the amount of IAA in the samples was expressed as μg IAA/mL/OD.

#### 4.3.4. Phosphate Solubilization

To determine the bacteria ability to solubilize phosphate, the method described by Nautiyal [90] with some modifications [89] was followed. Bacteria were grown in a modified Pikosvakaya medium, composed by 10 g of D-glucose, 5 g of tricalcium phosphate (Ca_3_(PO_4_)_2_) (Sigma), 0.5 g of ammonium sulphate ((NH_4_)_2_SO_4_) (Sigma), 0.2 g of NaCl (Sigma), 0.1 g of magnesium sulphate heptahydrate (MgSO_4_·7H_2_O) (Merck), 0.2 g of potassium chloride (KCl) (Merck), 0.5 g yeast extract (Alfa Aesar), 0.002 g of manganese sulphate monohydrate (MnSO4·H2O) (Sigma) and 0.002 g of iron (II) sulphate heptahydrate (FeSO_4_·7H_2_O) (Merck). After adjusting the pH to 7, 10 g of agar (Liofilchem) was added. The modified medium was sterilized in an autoclave for 20 min, at 120 °C. The bacteria were inoculated, and the plates were incubated for 10 days at 26 °C. After 10 days the colony and halo zone diameter (including the colony) were measured, allowing to calculate the solubilization index (SI = Halo diameter/Colony diameter).

### 4.4. Greenhouse Experiment

*Zea mays* seeds used in this work (certified trial seeds, Pioneer Optimum AQUAmax hybrids, variety P9911) were described as drought tolerant, and were kindly offered by Cooperativa Agrícola de Esposende and Pioneer.

*Zea mays* seeds were hydrated for 2 days in running water. The germinated seeds were sown in cups with washed and autoclaved river sand. The experiment included 2 conditions (watered on drought), each with 3 replicates (each cup was a replicate) with 3 seeds per cup. Each condition included a not-inoculated control (marked as Ctl) and inoculated with one of the 18 strains.

The maize was grown in a greenhouse, at 25 °C, with a photoperiod of 16:8. The 114 cups ((18 strains + 1 control) × 2 conditions × 3 replicates = 19 × 2 × 3 = 114) of the experiment were randomly distributed in a shelf. The luminous intensity hitting the surface sand was between 7500 and 9000 lux, measured with Testo 540. 

The cups were initially irrigated with 40 mL of distilled water (dH_2_O). The drought condition was only watered once, with 5 mL of dH_2_O, and the control was watered 2 times, with 15 mL of dH_2_O.The assay had the duration of 6 days. The shoots and roots of control (non-inoculated) and plants inoculated were measured, weighted and used immediately (photosynthetic pigments) or frozen (−20 °C) for future use. Seedling emergence was determined when the non-inoculated cups, under irrigation, reached 90% of germination (4th day of growth). Biochemical analysis was performed in the bacterial strains that promoted seedling emergence (A, D, F, G, Q, R, S and T).

#### Photosynthetic Pigments

Chlorophylls and carotenoids were quantified in a dark cold room. Shoots (0.1 g) were homogenized with a mortar and pestle using 2 mL of cold acetone (80%). The homogenate was pipetted to a microtube and centrifuged at 10,000× *g*, for 20 min at 4 °C. In a 96-well microplate 150 μL of sample supernatant and 150 μL of acetone (80%) were pipetted to each well. A blank (300 μL of 80% acetone) was prepared. The absorbance was measured at 470, 645, 663 and 750 nm. The amount of chlorophylls a and b and carotenoids was determined using the equations proposed by Wellburn and Lichtenthaler [91], and the results were expressed in µg/g FW.

### 4.5. Biochemical Analysis

#### 4.5.1. Extraction

The bacterial strains biomass grown in the presence and absence of PEG (4.2) was extracted in sodium phosphate buffer (50 mM sodium dihydrogen phosphate monohydrate; 50 mM disodium hydrogen phosphate dihydrate; 1 mM ethylenediaminetetraacetic acid disodium salt dihydrate (EDTA); 1% (*v*/*v*) Triton X-100; 1 mM dithiothreitol (DTT), pH 7.0). The volume (between 500 μL and 1500 μL) of the buffer used varied with the amount of cells (optical density of each sample). Microtubes were vortexed, and bacterial cells lysed in an ultrasonic probe for 30 s at 0.6 Hz, always keeping the samples in an ice bath to prevent overheating, and centrifuged at 10,000× *g* for 10 min at 4 °C. The supernatant was used to determine the activity of superoxide dismutase (SOD), catalase (CAT), glutathione S-Transferases (GST), protein content (PROT) and protein carbonylation (PC), and the pellet used for determination of lipid peroxidation. Both fractions were used immediately or frozen at −20 °C until used.

For proline extraction, the samples were suspended in 3% sulfosalicylic acid, lysed for 30 s in an ultrasonic probe and centrifuged at 12,000× *g* for 10 min at 4 °C. The supernatant was collected for further analysis.

Shoots of plants (0.2 g frozen material) grown in two water regimes and inoculated or not with a bacterial strain (2.4) were extracted with 0.5 mL of potassium phosphate buffer (50 mM sodium dihydrogen phosphate monohydrate; 50 mM disodium hydrogen phosphate dihydrate; 1 mM EDTA; 1% (*v*/*v*) Triton X-100; 1 mM DTT, pH 7.0) (1:2 *w*/*v*) and centrifuged at 10,000× *g* for 10 min at 4 °C. The supernatant and pellet were used immediately or frozen (−20 °C) until used. The supernatant was used to determine CAT, SOD, and electron transport system (ETS) activity, PC and PROT content. The pellet was used for determination of LPO.

For proline determination 0.2 g of frozen shoot was homogenized with 0.5 mL of 3% sulfosalicylic acid (1:2 *w*/*v*) and centrifuged at 10,000× *g* for 10 min at 4 °C. The supernatant was collected and frozen (−20 °C) until further use.

#### 4.5.2. Lipid Peroxidation

The quantification of thiobarbituric acid reactive substances (TBARS) was performed according to the procedure described by Buege and Aust [92], using the bacteria and plant pellets. To the pellet 200 μL of 0.5% 2-thiobarbituric acid (TBA) and 150 μL of 20% trichloroacetic acid (TCA) were added. The blank was also prepared (200 μL of TBA and 150 μL of TCA). The microtubes were pierced and incubated at 96 °C for 25 min, and the reaction was stopped by placing the samples in ice. To a 96-well microplate 250 μL of each microtube were transferred to each well and the absorbance read at 532 nm. The LPO was expressed in pmol of MDA equivalents per million cells in bacteria (pmol/M cells), and nmol of MDA equivalents per g of fresh weight (nmol/g FW) in plants.

#### 4.5.3. Superoxide Dismutase

The methodology used was based on the procedure described by Beauchamp and Fridovich [93]. In a 96-well microplate, to 25 μL of supernatant, 250 μL of reaction buffer (50 mM Tris-HCl (pH 8.0), 0.1 mM DTPA, 0.1 mM Hypoxanthine) with tetrazolium salt (NBT) and 25 μL of xanthine oxidase (XO), were added and incubated for 20 min at room temperature with orbital rotation. Blanks were also prepared (by 300 μL reaction buffer without NBT). The absorbance was measured at 560 nm and the results were expressed in μU per million cells (μU/million cells) in bacteria and U per g of fresh weight (U/g FW) in plants. One unit of enzymatic activity (U) refers to a 50% inhibition of NBT reduction.

#### 4.5.4. Catalase

This methodology was based on the one described by Johansson and Borg [94]. In a 96-well microplate was added 25 μL of sample, followed by 125 μL of reaction buffer (1 M K_2_HPO_4_ and 1 M KH_2_PO_4_, pH 7.0), 37.5 μL of methanol and 25 μL of 35.28 mM hydrogen peroxide (H_2_O_2_). After 20 min of incubation at room temperature, was added 37.5 μL of 10 M potassium hydroxide (KOH) and 37.5 μL of 34.2 mM Purpald, followed by 10 min of incubation at room temperature, and 12.5 μL of Potassium periodate (KIO_4_). After an incubation of 5 min, the absorbance was read at 540 nm. The catalase production was determined using a standard curve with increasing concentrations of formaldehyde (0–300 μL) and the results were expressed in μU/million cells in bacteria and mU/g FW in plants.

#### 4.5.5. Glutathione S-Transferases

The activity of glutathione S-transferases in bacteria was determined following an adaptation of the method described by Habig et al. [95]. Sequentially, on a 96-well microplate was added 100 μL of supernatant, 200 μL of reaction solution (0.1 M potassium phosphate buffer, pH 6.5, 10 mM GSH and 60 mM CNDB) and the absorbance was read at 340 nm during 15 min in intervals of 15 s. The results were expressed in U/million cells, where U represents the quantity of enzyme that catalyses 1 µmol of thioether formed per min.

#### 4.5.6. Protein

Protein was determined following the method described by Robinson and Hogden [96]. To a 96-well microplate was added 25 μL of sample and 275 μL of Biuret reagent. After 10 min of incubation in the dark, the absorbance was read at 540 nm and the final concentration of protein was calculated using a standard curve with increasing concentrations of BSA solution (0, 5, 10, 20, and 40 mg/mL). The results were expressed in mg/million cells in bacteria and mg/g FW in plants.

#### 4.5.7. Protein Carbonylation

Carbonyl groups in proteins were determined using the methods described by Mesquita et al. [97] and Udenigwe et al. [98]. Into a 96-well microplate was added 120 μL of sample or blank, followed by 120 μL of 2,4-dinitrophenylhydrazine (DNPH). After 10 min of incubation at room temperature, was added 60 μL of sodium hydroxide (NaOH) and the reaction was incubated during 10 min. The absorbance was read at 450 nm, and the results were expressed in μmol/million cells in bacteria and μmol/g of FW in plants.

#### 4.5.8. ETS

The ETS activity was measured based on the method described by Kind and Packard [99]. In a 96-well microplate was added 35.7 μL of supernatant, 107 μL of BSS buffer (0.13 M Tris-HCl and 0.3% Triton X-100, pH 8.5), 35.7 μL of NAD(P)H and 71.4 μL of 8 mM *p*-IodoNitroTetrazolium (INT). The absorbance was read at 490 nm during 15 min in 25 s intervals. The results were expressed in nmol/min per g of plant FW.

#### 4.5.9. Soluble Sugars

The determination of soluble sugars in plants was performed based on the method described by Dubois et al. [100]. In a microtube was added 10 μL of supernatant, 100 μL of 5% phenol and 600 μL of 98% sulfuric acid (H_2_SO_4_). After 30 min incubation at room temperature, 300 μL from each microtube were transferred to a 96-well microplate and the absorbance was read at 492 nm. The final concentration was calculated using a standard curve with increasing concentrations of glucose (0, 0.1, 0.4, 1, 2, 3, 4 and 5 mg/mL). The results were expressed in mg/g plant FW.

#### 4.5.10. Proline

The determination of proline content was performed following the method described by Bates et al. [101]. To 50 μL of sample were added 50 μL of acid ninhydrin and 50 μL of glacial acetic acid. The microtubes were pierced and incubated for 1 h at 100 °C, and the reaction was stopped by placing the samples in ice, and 125 μL of the samples were added to a 96-well microplate. The blanks used were constituted by 100 μL of sodium phosphate buffer, 100 μL of acid ninhydrin and 100 μL of glacial acetic acid. The absorbance was measured at 520 nm and proline standards (0–1.5 mg/mL) were used. Results were expressed in milligrams of proline per million cells (mg/million cells). 

### 4.6. Statistical Analysis

All parameters assessed were submitted to hypothesis testing. Parameters were analysed following a one-way hierarchical design, with PEG conditions (absence and presence) as fixed factor. The matrix resemblance was obtained using Euclidean distance. The effect of PEG on samples was determined using pair-wise Permutational Multivariate Analysis of Variance (PERMANOVA), using PRIMER version 6.1.16 for Windows.

For bacteria, the null hypothesis tested was for each bacterial strain no significant differences exist between control (no PEG) and IC_50_. For plants the null hypothesis assessed was no significant differences exist between non-inoculated (control) and inoculated plants under watered and drought conditions. Significant differences were considered for *p* ≤ 0.05 and were identified in figures with single asterisks (for *p* ≤ 0.05) and double asterisks (for *p* ≤ 0.01).

A matrix combining the variables (LPO, SOD, CAT, PC, PROT, proline, GST, ETS and soluble carbohydrates) and the difference between osmotic stress (PEG for bacteria, drought for plants) and control non-osmotically stressed was performed, and the Euclidean distance similarity matrix was calculated after data normalisation. The similarity matrix was submitted to ordination analysis, and a Principal Coordinates Ordination (PCO) was performed. Based on the Pearson correlation vectors of biomarkers obtained (r > 0.50 for bacteria and r > 0.80 for plants) it was possible to identify the biochemical parameters that imposed more differences when organisms (bacteria or plants) were exposed to stress.

## 5. Conclusions

The study of soil bacterial diversity in Africa is limited, and the research on topics like application of bioinoculants to promote plant growth is very scarce. This study gives information on the culturable bacteria associated to plants growing in arid zones conditions, and that help plants survive in environments with limited water availability. These bacteria could be used as effective inoculants helping crops cope with water shortage and reducing the impacts of drought on yields. Some strains were able to promote the root growth of drought stressed plants, through the production of phytohormones, siderophores and moderate phosphate solubilization. The induction of osmolytes, also emerged as a factor contributing to plant tolerance to drought. Finally, the influence of bacterial inoculation on the antioxidant response also bestowed plant tolerance to drought. Evidencing that this bacterial tripartite influence can further increase the tolerance of drought tolerant varieties, and can be of major value to protect plants from water scarcity, increase water management efficiency, minimize yield losses and contribute to food security.

## Figures and Tables

**Table 1:** Identification and plant growth-promoting abilities of bacteria isolated from Cape Verde. Determination of the percentage of polyethylene glycol that inhibits 50% of bacterial growth (IC50 PEG). Indole acetic acid (IAA) and alginate production were expressed in μg/mL/DO. Siderophore production was expressed in percent siderophore units per optical density (PSU/DO). Phosphate solubilization was expressed by the ratio between halo and colony diameter. The plant emergence rate was determined in the 4th day of growth, in comparison with not-inoculated control (↑— increase, ↓—decrease, ↔—no change).

Species	Accession Number	Code	Isolated from	IC50 PEG	Siderophores (PSU/OD)	Alginate (μg/mL/OD)	Phosphate Solubilization (Ratio)	IAA (μg/mL/OD)	Seedling Emergence
*Pantoea* sp.	OM985627	A	*Acacia albida*	7.5	22.04 ± 2.73	19.25 ± 1.01	1.18 ± 0.09	4.23 ± 0.48	↑
*Klebsiella* sp.	OM985630	B	*Acacia albida*	7.5	13.96 ± 4.39	19.75 ± 0.56	1.25 ± 0.07	5.60 ± 0.33	↔
*Pseudomonas* sp.	OM985635	D	*Acacia albida*	7.5	23.43 ± 4.25	24.88 ± 0.94	1.25 ± 0.14	4.44 ± 0.39	↑
*Pseudomonas* sp.	OM985638	E	*Acacia albida*	7.5	18.94 ± 3.14	23.42 ± 0.89	−	4.76 ± 0.43	↔
*Acinetobacter* sp.	OM985640	F	*Acacia albida*	7.5	32.23 ± 5.45	22.66 ± 0.59	1.71 ± 0.21	3.72 ± 0.52	↑
*Stenotrophomonas* sp.	OM985622	G	*Acacia albida*	7.5	16.12 ± 2.29	13.56 ± 0.80	1.03 ± 0.09	4.36 ± 0.45	↑
*Enterobacter* sp.	OM985625	H	*Acacia albida*	7.5	13.90 ± 8.64	15.79 ± 0.50	1.97 ± 0.19	4.75 ± 0.46	↔
*Enterobacter* sp.	OM985623	J	*Amaranthus viridis*	7.5	25.83 ± 1.80	18.27 ± 2.72	1.88 ± 0.27	9.62 ± 0.83	↔
*Pantoea* sp.	OM985626	K	*Amaranthus viridis*	7.5	22.05 ± 7.83	25.55 ± 0.94	1.32 ± 0.12	9.87 ± 1.23	↔
*Pseudomonas* sp.	OM985629	L	*Amaranthus viridis*	7.5	57.54 ± 4.89	15.07 ± 1.82	1.40 ± 0.07	4.68 ± 1.33	↔
*Rhizobium* sp.	OM985632	M	*Acacia albida*	7.5	22.54 ± 5.76	14.12 ± 1.71	1.08 ± 0.14	4.17 ± 0.52	↔
*Paenarthrobacter* sp.	OM985634	N	*Acacia albida*	12.5	49.94 ± 4.64	7.43 ± 0.76	−	5.71 ± 0.80	↔
*Ochrobactrum* sp.	OM985637	O	*Acacia albida*	12.5	17.77 ± 0.96	12.84 ± 0.82	1.94 ± 0.14	6.15 ± 0.80	↓
*Pseudomonas* sp.	OM985621	Q	*Acacia albida*	10	15.77 ± 5.69	15.94 ± 3.31	1.04 ± 0.03	6.39 ± 0.88	↑
*Rhizobium* sp.	OM985624	R	*Acacia albida*	10	23.29 ± 8.10	14.71 ± 1.66	1.15 ± 0.03	4.67 ± 0.34	↑
*Stenotrophomonas* sp.	OM985631	S	*Acacia albida*	7.5	51.83 ± 5.77	25.16 ± 4.08	−	7.23 ± 0.41	↑
*Pseudomonas* sp.	OM985633	T	*Acacia albida*	10	12.04 ± 3.77	13.24 ± 1.51	1.38 ± 0.24	4.34 ± 0.61	↑
*Enterobacter* sp.	OM985636	U	*Acacia albida*	10	13.91 ± 1.32	19.67 ± 2.94	1.88 ± 0.17	3.80 ± 0.57	↔

## Data Availability

Not applicable.

## Supplementary Materials

The following supporting information can be downloaded at: https://www.mdpi.com/article/10.3390/plants11212912/s1, Table S1: Results obtained for biochemical parameters of bacterial strains grown in osmotic stress (% PEG inhibiting growth 50%) and control (no PEG added); Table S2: Maize plants grown for 7 days in watered and drought conditions. Morphometric parameters (fresh weight and length of plants) and variation of photosynthetic pigments in inoculated (A, D, F, G, Q, R, S, T) and non-inoculated (control–Ctl) plants; Table S3: Maize plants grown for 7 days in watered and drought conditions. Biochemical parameters evaluated in inoculated (A, D, F, G, Q, R, S, T strains) and non-inoculated (control–Ctl) plants.
