# Supplementary material for: Bioprospecting Soil Bacteria from Arid Zones to Increase Plant Tolerance to Drought: Growth and Biochemical Status of Maize Inoculated with Plant Growth-Promoting Bacteria Isolated from Sal Island, Cape Verde
Source: Plants (Basel). 2022 Oct 29;11(21):2912. doi: 10.3390/plants11212912 (PMC9656834; doi:10.3390/plants11212912)
Supplement: Supplementary file 1 [file plants-11-02912-s001.zip › plants-1968574-supplementary.pdf]

Table S1: Results obtained for biochemical parameters of bacterial strains grown in osmotic stress (% PEG inhibiting growth 50%) and control (no PEG added). Bacterial strains: *Pantoea spp*. (A); *Klebsiella spp*. (B); *Pseudomonas spp*. (D); *Pseudomonas spp*. (E); *Acinetobacter spp*. (F); *Stenotrophomonas spp*. (G); *Enterobacter spp*. (H); *Enterobacter spp*. (J); *Pantoea spp*. (K); *Pseudomonas spp*. (L); *Rhizobium spp*. (M); *Paenarthrobacter spp*. (N); *Ochrobactrum spp*. (O); *Pseudomonas spp*. (Q); *Rhizobium spp*. (R); *Stenotrophomonas spp*. (S); *Pseudomonas spp*. (T); *Enterobacter spp*. (U). Values are means of three replicates + standard deviation. Statistical analysis was performed relatively to non-inoculated control. Significant differences were considered for $p \leq 0.05$ and were identified with values in bold and single asterisks (for $p \leq 0.05$) and double asterisks (for $p \leq 0.01$).

	LPO (pmoles/M cells)		SOD (µU/M cells)		CAT (µU/M cells)		Proline (µg/M cells)		GST (µU/M cells)		PC (mmol/M cells)		PROT (µg prot/M cells)	
	control	PEG	control	PEG	control	PEG	control	PEG	control	PEG	control	PEG	control	PEG
**A**	1.45 ± 0.32	**4.51 ± 0.54****	111.30 ± 1.40	**136.21 ± 2.39****	0.32 ± 0.10	2.01 ± 0.60	3.44 ± 0.36	5.97 ± 1.14	3.62 ± 0.02	**4.02 ± 0.11***	0.30 ± 0.04	**0.48 ± 0.06***	0.29 ± 0.09	0.43 ± 0.01
**B**	1.46 ± 0.29	**4.62 ± 1.16***	116.94 ± 2.06	123.95 ± 7.48	1.23 ± 0.25	**5.16 ± 1.68***	4.00 ± 0.56	4.93 ± 0.23	3.36 ± 0.15	3.37 ± 0.18	0.46 ± 0.07	0.38 ± 0.04	0.16 ± 0.04	**0.33 ± 0.10***
**D**	0.73 ± 0.28	**1.79 ± 0.23****	120.82 ± 5.76	**142.78 ± 12.24***	1.33 ± 0.11	**2.44 ± 0.06****	4.26 ± 0.28	**5.89 ± 0.57***	4.04 ± 0.21	3.90 ± 0.36	0.51 ± 0.12	0.52 ± 0.07	0.44 ± 0.19	0.40 ± 0.08
**E**	0.77 ± 0.04	**3.33 ± 0.81****	119.54 ± 11.65	138.86 ± 10.56	1.31 ± 0.12	1.28 ± 0.25	7.21 ± 0.81	7.59 ± 1.57	3.99 ± 0.21	3.89 ± 0.21	0.46 ± 0.09	0.46 ± 0.10	0.33 ± 0.09	0.27 ± 0.03
**F**	1.29 ± 0.20	1.84 ± 0.43	111.94 ± 3.37	135.24 ± 17.86	1.10 ± 0.12	1.53 ± 0.41	4.07 ± 0.97	5.30 ± 0.84	3.45 ± 0.24	3.62 ± 0.32	0.29 ± 0.03	**0.49 ± 0.12***	0.23 ± 0.04	**0.59 ± 0.14***
**G**	1.04 ± 0.23	**6.19 ± 1.42****	138.17 ± 11.16	145.83 ± 4.33	1.45 ± 0.46	**5.39 ± 1.64***	4.63 ± 0.18	6.11 ± 1.45	4.27 ± 0.44	3.76 ± 0.22	0.38 ± 0.05	**0.48 ± 0.01***	0.23 ± 0.03	**0.28 ± 0.01***
**H**	1.36 ± 0.20	**3.79 ± 0.44****	131.57 ± 1.31	119.14 ± 8.20	1.50 ± 0.11	**5.43 ± 1.77***	5.74 ± 0.85	4.84 ± 0.68	4.09 ± 0.51	3.28 ± 0.26	0.45 ± 0.10	0.46 ± 0.05	0.18 ± 0.06	0.26 ± 0.06
**J**	1.49 ± 0.60	**4.39 ± 0.70****	123.83 ± 1.21	158.27 ± 23.66	1.86 ± 0.78	1.22 ± 0.18	5.31 ± 0.67	7.06 ± 1.15	4.52 ± 0.67	4.62 ± 0.59	0.54 ± 0.09	0.64 ± 0.15	0.33 ± 0.07	0.27 ± 0.07
**K**	1.22 ± 0.18	**0.10 ± 0.02****	129.43 ± 27.13	201.44 ± 45.91	1.29 ± 0.40	**2.39 ± 0.16***	5.50 ± 1.51	9.60 ± 1.29	3.37 ± 0.54	**7.82 ± 0.30****	0.43 ± 0.03	**0.89 ± 0.03****	0.23 ± 0.08	0.29 ± 0.05
**L**	1.01 ± 0.17	**0.08 ± 0.02****	156.63 ± 19.16	**44.13 ± 14.03****	1.25 ± 0.16	**14.37 ± 5.51***	4.74 ± 0.67	**6.75 ± 1.31***	3.33 ± 0.68	**5.42 ± 0.19****	0.37 ± 0.11	**0.67 ± 0.08***	0.30 ± 0.03	**0.49 ± 0.10***
**M**	1.34 ± 0.25	**0.04 ± 0.01****	140.72 ± 2.19	166.68 ± 36.23	1.36 ± 0.43	**14.18 ± 4.77****	5.40 ± 0.25	6.22 ± 0.63	4.32 ± 0.17	4.83 ± 0.47	0.48 ± 0.10	0.59 ± 0.07	0.45 ± 0.08	**0.25 ± 0.07***
**N**	2.48 ± 0.86	**0.06 ± 0.01****	115.25 ± 6.66	151.74 ± 33.89	2.48 ± 0.56	3.80 ± 1.30	7.20 ± 2.23	10.42 ± 2.43	4.19 ± 0.67	6.64 ± 1.53	0.50 ± 0.11	**0.73 ± 0.06***	0.87 ± 0.71	1.2 ± 0.50
**O**	0.44 ± 0.07	1.08 ± 0.42	130.43 ± 13.56	116.46 ± 17.01	2.12 ± 0.53	1.58 ± 0.59	5.14 ± 0.57	5.21 ± 1.27	3.88 ± 0.18	3.48 ± 0.66	0.46 ± 0.04	0.40 ± 0.07	0.31 ± 0.12	0.39 ± 0.14
**Q**	1.59 ± 0.42	2.07 ± 0.50	137.68 ± 6.63	102.76 ± 83.51	1.68 ± 0.26	**19.42 ± 5.57****	5.09 ± 0.34	5.92 ± 0.94	3.56 ± 0.53	3.06 ± 0.39	0.31 ± 0.04	0.38 ± 0.05	0.13 ± 0.03	**0.37 ± 0.09***
**R**	1.99 ± 0.35	1.86 ± 0.47	140.55 ± 6.45	**105.28 ± 18.45***	1.12 ± 0.12	**7.23 ± 2.25****	**5.93 ± 0.56***	4.72 ± 0.22	3.84 ± 0.34	3.40 ± 0.47	0.53 ± 0.10	0.41 ± 0.09	0.22 ± 0.06	0.52 ± 0.18
**S**	1.34 ± 0.24	**12.10 ± 2.21****	115.63 ± 17.49	62.53 ± 55.20	1.36 ± 0.25	**14.57 ± 5.13***	8.74 ± 0.78	**15.82 ± 3.61***	4.32 ± 0.58	**6.05 ± 0.40***	0.54 ± 0.08	0.49 ± 0.11	0.18 ± 0.02	**0.39 ± 0.10***
**T**	1.38 ± 0.24	**2.91 ± 0.78***	139.02 ± 12.91	**13.05 ± 3.44****	1.61 ± 0.19	**4.72 ± 1.75***	5.20 ± 0.08	5.71 ± 0.40	3.85 ± 0.13	3.90 ± 0.11	0.36 ± 0.05	0.47 ± 0.06	0.79 ± 0.14	**0.29 ± 0.03****
**U**	0.62 ± 0.16	**1.86 ± 0.42***	137.48 ± 8.61	118.35 ± 20.35	1.45 ± 0.16	**22.91 ± 4.57***	4.52 ± 0.19	**7.43 ± 1.33***	3.72 ± 0.44	4.31 ± 0.70	0.43 ± 0.12	0.57 ± 0.13	0.64 ± 0.31	0.53 ± 0.10

Table S2: Maize plants grown for 7 days in watered and drought conditions. Morphometric parameters (fresh weight and length of plants) and variation of photosynthetic pigments in inoculated (A, D, F, G, Q, R, S, T) and non-inoculated (control – Ctl) plants. Values are means of three replicates + standard deviation. Statistical analysis was performed relatively to non-inoculated watered control. Significant differences were considered for $p \leq 0.05$ and were identified with values in bold with single asterisks (for $p \leq 0.05$) and double asterisks (for $p \leq 0.01$). Bacterial strains: *Pantoea spp.* (A); *Pseudomonas spp*. (D); *Acinetobacter spp*. (F); *Stenotrophomonas spp*. (G); *Pseudomonas spp*. (Q); *Rhizobium spp*.(R); *Stenotrophomonas spp*. (S); *Pseudomonas spp*. (T).

	**Shoot Fresh Weight (g)**		**Root Fresh Weight (g)**		**Shoot lenght (cm)**		**Root lenght (cm)**		**Chl a (µg/g FW)**		**Chl b (µg/g FW)**		**Carotenoids (µg/g FW)**	
	**watered**	**drought**	**watered**	**drought**	**watered**	**drought**	**watered**	**drought**	**watered**	**drought**	**watered**	**drought**	**watered**	**drought**
**Ctl**	0.32 ± 0.02	0.30 ± 0.02	1.91 ± 0.15	**1.26 ± 0.10****	9.00 ± 0.33	9.28 ± 0.69	8.08 ± 8.39	8.39 ± 0.25	378.78 ± 108.73	444.62 ± 115.52	196.83 ± 61.22	**115.18 ± 22.95***	152.35 ± 34.82	**102.31 ± 20.32***
**A**	0.32 ± 0.04	0.29 ± 0.02	**1.49 ± 0.06***	**1.29 ± 0.08****	9.33 ± 1.15	8.72 ± 0.48	8.33 ± 0.33	**9.78 ± 0.84***	331.00 ± 133.64	527.22 ± 86.59	165.56 ± 69.71	138.14 ± 20.77	146.99 ± 51.16	128.01 ± 16.38
**D**	0.35 ± 0.03	0.26 ± 0.05	1.65 ± 0.22	**1.12 ± 0.16****	8.08 ± 0.65	8.31 ± 0.63	7.78 ± 0.77	8.11 ± 0.69	274.78 ± 118.33	**611.11 ± 136.06***	134.10 ± 52.64	156.33 ± 33.46	119.49 ± 47.64	137.77 ± 30.19
**F**	0.32 ± 0.06	0.27 ± 0.03	1.91 ± 0.26	**1.26 ± 0.09****	8.61 ± 0.67	**7.67 ± 0.44***	8.44 ± 0.69	8.44 ± 0.59	275.59 ± 116.35	276.87 ± 188.59	135.92 ± 50.37	**77.22 ± 51.99***	113.45 ± 45.52	**73.88 ± 51.53***
**G**	0.32 ± 0.05	0.30 ± 0.01	1.60 ± 0.30	**1.07 ± 0.07****	8.89 ± 0.51	8.67 ± 0.43	7.67 ± 0.58	8.00 ± 0.58	302.32 ± 87.19	464.26 ± 133.41	147.16 ± 40.40	120.04 ± 31.46	135.81 ± 29.09	104.73 ± 28.71
**Q**	0.34 ± 0.06	0.26 ± 0.05	**1.46 ± 0.15***	**1.37 ± 0.16***	8.83 ± 0.67	**7.50 ± 0.50***	8.33 ± 1.01	7.22 ± 0.69	372.24 ± 49.19	365.96 ± 251.04	176.54 ± 27.82	98.88 ± 55.79	157.37 ± 20.30	91.02 ± 51.12
**R**	0.33 ± 0.05	0.31 ± 0.00	1.55 ± 0.31	**1.56 ± 0.12***	8.39 ± 0.79	8.94 ± 0.42	7.33 ± 0.44	8.33 ± 0.58	286.93 ± 95.30	366.62 ± 148.16	138.09 ± 40.96	**114.81 ± 19.79***	127.27 ± 31.73	115.68 ± 12.81
**S**	0.33 ± 0.06	0.29 ± 0.03	1.95 ± 0.15	**1.46 ± 0.20***	9.17 ± 0.76	**8.01 ± 0.36***	8.22 ± 0.51	**9.34 ± 0.59***	411.88 ± 40.07	475.32 ± 172.64	204.15 ± 23.01	137.57 ± 27.19	179.00 ± 27.84	139.17 ± 29.15
**T**	0.31 ± 0.05	0.33 ± 0.03	**1.42 ± 0.19***	1.78 ± 0.20	8.17 ± 0.93	9.15 ± 0.83	7.83 ± 0.44	8.49 ± 1.20	**220.00 ± 60.98***	642.16 ± 185.62	**111.52 ± 29.50***	159.95 ± 47.48	**97.33 ± 20.92***	146.56 ± 32.17

Table S3: Maize plants grown for 7 days in watered and drought conditions. Biochemical parameters evaluated in inoculated (A, D, F, G, Q, R, S, T strains) and non-inoculated (control – Ctl) plants. Values are means of three replicates + standard deviation. Statistical analysis was performed relatively to non-inoculated (control – Ctl) watered plants. Significant differences were considered for $p \leq 0.05$ and were identified with values in bold and single asterisks (for $p \leq 0.05$) and double asterisks (for $p \leq 0.01$). Bacterial strains: *Pantoea spp.* (A); *Pseudomonas spp*. (D); *Acinetobacter spp*. (F); *Stenotrophomonas spp*. (G); *Pseudomonas spp*. (Q); *Rhizobium spp*.(R); *Stenotrophomonas spp*. (S); *Pseudomonas spp*. (T).

	**LPO (nmoles/g FW)**		**SOD (mU/g FW)**		**CAT (mU/g FW)**		**Proline (mg/g FW)**		**ETS (nmol/min * g FW)**		**PC (µmol/g FW)**		**PROT (mg prot/g FW)**		**Sugars (mg/g FW)**	
	**watered**	**drought**	**watered**	**drought**	**watered**	**drought**	**watered**	**drought**	**watered**	**drought**	**watered**	**drought**	**watered**	**drought**	**watered**	**drought**
**Ctl**	3.56 ± 0.93	2.76 ± 0.41	0.29 ± 0.04	**0.19 ± 0.04***	35.45 ± 7.11	22.66 ± 6.06	68.47 ± 15.14	100.17 ± 21.61	21.58 ± 4.15	14.25 ± 3.26	1.28 ± 0.36	0.92 ± 0.14	66.62 ± 9.03	81.12 ± 8.30	18.26 ± 4.23	15.57 ± 3.43
**A**	5.91 ± 1.38	2.89 ± 0.56	0.33 ± 0.06	0.24 ± 0.05	**49.46 ± 3.73***	**20.81 ± 4.08***	**113.09 ± 18.06***	85.17 ± 13.10	16.99 ± 3.38	13.91 ± 2.87	1.83 ± 0.59	0.92 ± 0.19	95.61 ± 16.01	70.17 ± 12.70	20.45 ± 1.61	13.14 ± 0.61
**D**	3.55 ± 0.57	3.25 ± 0.47	0.34 ± 0.04	**0.19 ± 0.02***	26.47 ± 4.02	**22.76 ± 0.77***	**108.75 ± 10.06***	**98.87 ± 8.90***	16.37 ± 3.70	**11.43 ± 1.91***	0.84 ± 0.27	**0.56 ± 0.13***	78.17 ± 16.80	65.59 ± 15.24	17.02 ± 3.45	18.21 ± 3.50
**F**	4.49 ± 0.81	2.96 ± 0.18	0.21 ± 0.05	0.27 ± 0.04	32.36 ± 11.02	25.39 ± 2.15	69.89 ± 22.11	83.35 ± 18.52	23.28 ± 3.63	16.39 ± 3.94	1.04 ± 0.15	**0.43 ± 0.06***	71.11 ± 7.98	80.34 ± 25.85	24.41 ± 6.43	13.71 ± 3.12
**G**	2.38 ± 0.64	2.79 ± 0.55	**0.18 ± 0.04***	0.22 ± 0.11	23.77 ± 6.67	25.15 ± 2.76	63.99 ± 10.66	93.54 ± 8.55	13.74 ± 2.97	**12.90 ± 2.12***	0.97 ± 0.25	**0.45 ± 0.10***	73.58 ± 11.84	69.38 ± 15.36	16.98 ± 3.57	13.32 ± 3.05
**Q**	2.59 ± 0.67	5.18 ± 0.62	0.21 ± 0.03	0.36 ± 0.13	**22.29 ± 1.09***	31.55 ± 8.54	78.85 ± 12.62	**141.51 ± 21.62***	15.91 ± 2.19	16.00 ± 2.05	**0.63 ± 0.16***	**0.59 ± 0.09***	95.74 ± 17.65	**137.32 ± 36.57***	**10.55 ± 1.28***	19.96 ± 4.64
**R**	2.34 ± 0.76	5.85 ± 1.49	**0.12 ± 0.03***	0.19 ± 0.12	**18.64 ± 3.57***	42.65 ± 9.86	71.20 ± 11.97	**162.40 ± 29.63***	17.58 ± 0.77	26.64 ± 5.11	**0.54 ± 0.08***	0.68 ± 0.25	62.63 ± 8.81	**134.06 ± 19.99***	18.70 ± 1.04	**37.34 ± 7.34***
**S**	4.01 ± 0.95	4.09 ± 0.85	0.21 ± 0.03	**0.16 ± 0.04***	30.03 ± 5.54	50.73 ± 11.14	92.86 ± 13.24	**128.90 ± 16.37***	23.90 ± 6.05	19.78 ± 2.24	**0.59 ± 0.08***	0.94 ± 0.08	69.36 ± 20.25	**91.02 ± 6.83***	19.43 ± 4.46	21.04 ± 3.65
**T**	3.47 ± 0.36	**5.54 ± 0.76***	0.34 ± 0.05	0.40 ± 0.11	25.92 ± 1.39	40.58 ± 13.41	95.96 ± 11.75	**134.39 ± 18.27***	19.28 ± 1.86	24.04 ± 2.92	1.22 ± 0.16	1.00 ± 0.17	106.69 ± 38.20	**152.57 ± 24.92***	24.24 ± 3.75	**31.62 ± 3.93***
